# Mass spectrometry-based assay for the molecular diagnosis of glioma: concomitant detection of chromosome 1p/19q codeletion, and *IDH1*, *IDH2*, and *TERT* mutation status

**DOI:** 10.18632/oncotarget.19103

**Published:** 2017-07-08

**Authors:** Chiara Pesenti, Leda Paganini, Laura Fontana, Emanuela Veniani, Letterio Runza, Stefano Ferrero, Silvano Bosari, Maura Menghi, Giovanni Marfia, Manuela Caroli, Rosamaria Silipigni, Silvana Guerneri, Silvia Tabano, Monica Miozzo

**Affiliations:** ^1^ Department of Pathophysiology and Transplantation, Università degli Studi di Milano, Milan, Italy; ^2^ Division of Pathology, Fondazione IRCCS Ca’ Granda Ospedale Maggiore Policlinico, Milan, Italy; ^3^ Department of Biomedical, Surgical and Dental Sciences, Università degli Studi di Milano, Milan, Italy; ^4^ Diatech Pharmacogenetics, Jesi, Italy; ^5^ Laboratory of Experimental Neurosurgery and Cell Therapy, Neurosurgery Unit, Fondazione IRCCS Ca’ Granda, Ospedale Maggiore Policlinico, Milan, Italy; ^6^ Neurosurgery Unit, Fondazione IRCCS Ca' Granda, Ospedale Maggiore Policlinico, Milan, Italy; ^7^ Laboratory of Medical Genetics, Fondazione IRCCS Ca' Granda Ospedale Maggiore Policlinico, Milan, Italy

**Keywords:** glioma, 1p/19q LOH, massARRAY, IDH, TERT

## Abstract

The World Health Organization recently revised the diagnosis of glioma, to integrate molecular parameters, including IDH mutations and codeletion (loss of heterozygosity; LOH) of chromosome arms 1p/19q, into the definitions of adult glioma histological subtypes. Mutations in the *TERT* promoter may also be useful for glioma diagnosis and prognosis. The integration of molecular markers into routine diagnosis requires their rapid and reliable assessment. We propose a MassARRAY (MS)-based test that can identify 1p/19q codeletion using quantitative SNP genotyping and, simultaneously, characterize hotspot mutations in the *IDH1*, *IDH2*, and *TERT* genes in tumor DNA. We determined the reliability of the MS approach testing 50 gliomas and comparing the MS results with those obtained by standard methods, such as short tandem repeat genotyping, array comparative genomic hybridization (array-CGH) and Fluorescence In Situ Hybridization (FISH) for 1p/19q codeletion and Sanger sequencing for hotspots mutations. The results indicate that MS is suitable for the accurate, rapid, and cost-effective evaluation of chromosome deletions combined with hotspot mutation detection. This MS approach could be similarly exploited in evaluation of LOH in other situations of clinical and/or research importance.

## INTRODUCTION

Diffuse gliomas are the most common brain tumor, accounting for 27% of all brain neoplasms and 80% of malignant tumors [1]. Until last year, diffuse gliomas were classified on the basis of histological criteria and 2007 World Health Organization (WHO) grading [2]. The increasing and extensive characterization of the genomic landscape of gliomas prompted the identification of genetic and epigenetic markers useful for tumor molecular classification; in the future, these molecular signatures could also represent actionable targets in gliomas, as is already the case for other cancers [3–5].

At the beginning of 2016, the WHO Classification of Tumors of the Central Nervous System (CNS) (2016 CNS WHO) revised the diagnostic guidelines for gliomas, to include molecular markers and create a novel concept of diagnosis, termed “integrated” diagnosis, characterized by the concomitant evaluation of phenotypic and genotypic parameters [6]. This new integrated diagnostic process not only aims to achieve greater objectivity, but also to improve patient management. The 2016 CNS WHO criteria state that, for a complete diagnosis of the types of adult glioma, analysis for specific mutations of the two genes, *IDH1* and *IDH2* (IDH), which encode isocitrate dehydrogenase 1 and 2, respectively, and of chromosome 1p/19q codeletion status, is essential.

Mutations of *IDH1* (codon 132) or *IDH2* (codon 172) allow discrimination between two classes of glioblastoma (GBM), IDH-wildtype and IDH-mutant, with differing genomic and epigenomic landscapes and prognoses [5, 7, 8]. Moreover, IDH mutations are almost invariably present in low grade gliomas (LGGs), including oligodendroglioma (ODG) and astrocytoma (AC) [7, 9]. IDH mutations are an early event in glioma tumorigenesis [5], and several mechanisms have been proposed to explain their role in neoplastic transformation. Mutant IDH enzymes acquire neomorphic activity and produce an oncometabolite, 2-HG, which is able to modify the epigenetic profile of cells, leading to the establishment of the CpG island methylator phenotype (G-CIMP). The production of 2-HG stimulates cell proliferation by reducing levels of the hypoxia-inducible factors. Moreover, IDH mutations promote tumorigenesis by decreasing intracellular levels of NADPH, which also has the effect of increasing the sensitivity of tumor cells to cytotoxic therapies, accounting for the positive prognostic value of this marker (see [10] for a review).

Once the IDH mutation status has been defined, the histological type of LGGs can be determined by evaluation of 1p/19q status. Codeletion of the chromosome arms 1p and 19q (1p/19q loss of heterozygosity; LOH) is the result of the unbalanced translocation [t(1;19)(q10;p10)] and enables discrimination between ODG and AC. Both of these LGGs are characterized by IDH mutations; however, only ODGs exhibit 1p/19q codeletion [6, 9]. Furthermore, the presence of 1p/19q LOH in ODGs is positively correlated with sensitivity to radiotherapy and chemotherapy using alkylating agents [11].

In addition to IDH and 1p/19q status, two mutations in the promoter region of the telomerase reverse transcriptase (*TERT*) gene are frequently identified in various types of cancer [12, 13], including glioma [14, 15]. These single nucleotide substitutions occur in a mutually exclusive manner in two hotspot positions upstream of the ATG start site: c.-124G>A and c.-146G>A (also termed C228T and C250T, respectively). The mutations generate a consensus binding site for the E-twenty-six transcription factor, which upregulates *TERT* expression and thus induces the maintenance of telomere length and tumor proliferation [16–19].

*TERT* promoter mutations are found in more than 70% of GBMs, in particular in the IDH-wildtype subgroup, and in almost all ODGs (nearly 95%) [6]. These mutations occur less frequently in ACs, which usually have mutations in the alpha thalassemia/mental retardation syndrome X-linked (*ATRX*) gene, another locus involved in telomere lengthening [20]. The presence of *TERT* mutations is also important for glioma prognosis, since gliomas (in particular LGGs) with concomitant IDH and *TERT* mutations are associated with better prognosis, while patients with IDH-wildtype and *TERT* mutated GBMs have poorer prognoses.

The absence of *MGMT* promoter methylation is an additional negative prognostic factor in *TERT* mutated GBM [8, 15, 21–24], and assessment of *MGMT* promoter methylation status assists in better defining prognosis for patients with gliomas, due to the role of this feature as a predictive marker of therapeutic efficacy [25].

**Figure 1:**
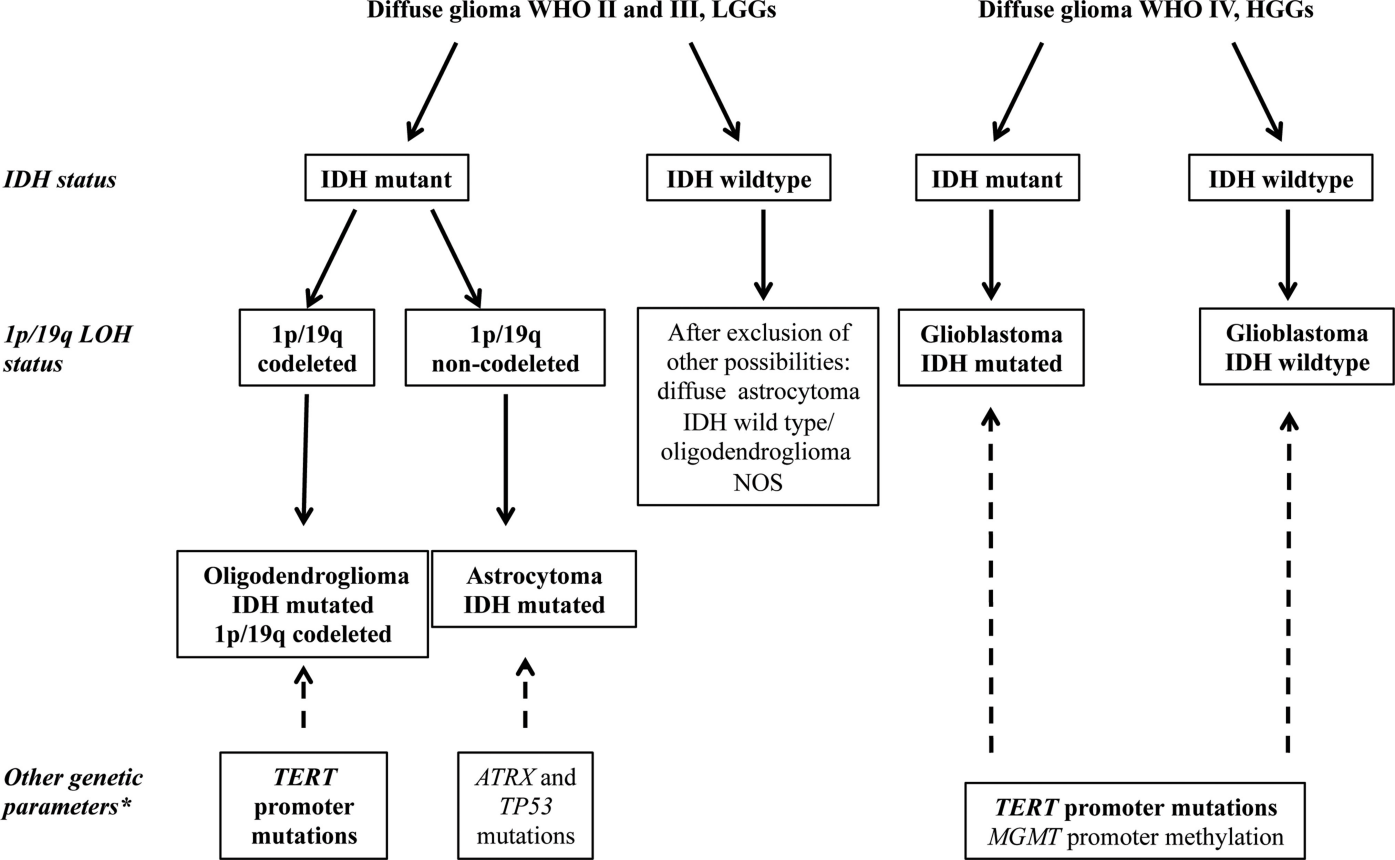
Simplified flowchart for the molecular characterization of diffuse gliomas (adapted from 2016 CNS WHO [5]) The detection of IDH mutations allows the distinction between primary and secondary GBM in HGGs and is necessary to discriminate between the LGGs, astrocytoma, and oligodendroglioma. LGGs with wildtype IDH are very rarely observed and, after the exclusion of other possible diagnoses, should be classified as NOS (not otherwise specified). The analysis of 1p/19q LOH is necessary to distinguish between oligodendroglioma and astrocytoma. *Other genetic markers characteristic of each type of glioma, not yet required for diagnosis, but useful for the molecular characterization of glioma (i.e., *TERT*, *TP53*, and *ATRX* mutations, and *MGMT* methylation).

Figure 1 summarizes the molecular diagnostic flowchart proposed by the 2016 CNS WHO, comprising analysis of the markers described above, and suggests the evaluation of additional genes, including *ATRX* and *TP53* mutations [4, 20], for profiling of ACs, *TERT* mutations for ODGs and GBMs, and *MGMT* promoter methylation for GBMs.

Together, these molecular markers allow the stratification of glioma patients in terms of prognosis and response to treatments [20, 26, 27], highlighting the importance of early molecular profiling of gliomas for a more precise patient management.

The presence of 1p/19q LOH is routinely assessed by FISH or genotyping of short tandem repeats (STRs), while IDH and *TERT* mutations are usually investigated by sequencing or real-time PCR. The use of multiple techniques for diagnostic purposes is laborious and time-consuming. Conversely, next generation sequencing (NGS) approaches [28–30] allow the simultaneous detection of markers; however, the technique is very expensive for use in medium-sized laboratories where, in general, only a few cases will be analyzed simultaneously. To overcome these limitations, we propose the MS system as a feasible high-throughput technology to simultaneously define the presence of 1p/19q LOH and hotspot mutations in *IDH1*, *IDH2*, and *TERT*.

MS technology uses a matrix-assisted laser desorption ionization time-of-flight (MALDI-TOF) mass-spectrometry platform to perform multiplex genotyping with high accuracy, even where only small amounts of poor-quality template material, such as DNA obtained from formalin-fixed paraffin-embedded (FFPE) tissue samples, are available [31]. The technique has already been introduced for routine diagnostics as a fast, reliable, and cost-effective approach, with specific CE marked *in vitro* diagnostic tests in Europe for the molecular characterization of colon and lung cancer [32]. Two previous reports [33, 34] investigated colon and lung cancers and demonstrated that MS can be exploited for the identification of chromosome deletions. In particular, van Puijgenbroek et al. [33] analyzed only one SNP in colon cancers and Tai et al. [34] evaluated a panel of SNPs, but without a quantitative approach to define the presence/absence of the deletions. Nevertheless, deletion analysis by MS has not been previously applied in molecular diagnosis of cancers, and CE marked *in vitro* diagnostic tests using this method are not currently available to analyze LOH, either alone or together with cancer hotspot mutations.

To develop an accurate test for 1p/19q LOH by MS, we quantitatively genotyped a panel of highly polymorphic biallelic single nucleotide polymorphisms (SNPs) evenly distributed along chromosomes 1p and 19q. We applied the proposed technique to 50 gliomas and compared the results of MS for 1p/19q LOH with those obtained by the standard methods, STR genotyping, array-CGH and FISH. In addition, we genotyped by MS *IDH1* codon 132, *IDH2* codon 172, *TERT* c.-124C>T and c.-146C>T promoter mutations, validating the results by Sanger sequencing.

The results described here demonstrate that the MS test accurately combines the evaluation of IDH and *TERT* mutations with that of 1p/19q LOH, in accordance with the recent WHO guidelines.

## RESULTS

### MS assay optimization for molecular diagnosis of gliomas

The MS assay optimized in our laboratory is able to reveal LOH at 1p/19q chromosome arms and the presence of hotspot mutations in *IDH1*, *IDH2*, and *TERT*. It was optimized and validated by the analysis of 50 glioma samples, as detailed below.

**Figure 2:**
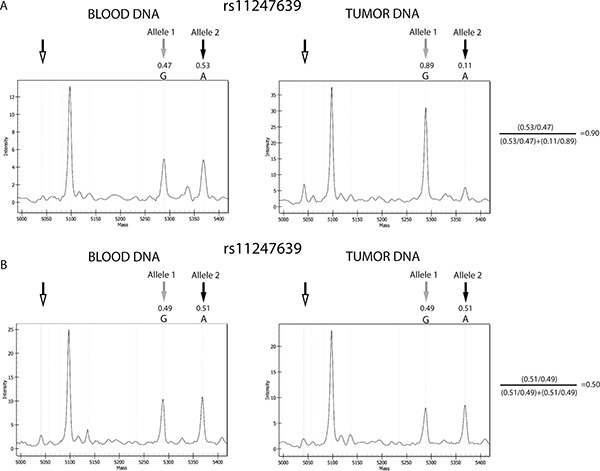
Example of results from gliomas with and without 1p/19q codeletion Comparison between the allele signals from the informative SNP, rs11247639, from blood and tumor DNA from sample 10 and 28. (**A**) Spectra from a sample positive for LOH. Tumor DNA shows evident reduction of the allele 2 signal, compared with blood DNA. (**B**) Spectra from a sample negative for LOH. No relevant differences were observed in allele frequencies between blood and tumor DNA. Black and gray arrows indicate the Allele 1 and 2 peaks, respectively, while the unfilled arrows indicate the positions of the extension primers. Allele frequencies calculated by the MS software are shown, and the application of the formula confirming the presence or absence of LOH is presented to the right of the plots. The other peak present in the spectra represents the allele signal of another SNP of the MS test. The results from other informative SNPs in samples 10 and 28 were concordant with those from rs11247639 and confirmed the presence of codeletion in case 10 and its absence in case 28.

For 1p/19q LOH analysis, we applied specific criteria to define SNPs as heterozygous and a formula to assess LOH, as detailed in Materials and Methods. An example of a sample with LOH, in which one of the two alleles detected in blood DNA was lost in the corresponding tumor DNA, thus significantly modifying the allele frequencies and resulting in a value of 0.9 using our formula, is presented in Figure 2A. An example of a sample with no LOH, in which the allele signals and frequencies from peripheral blood lymphocyte (PBL) and tumor DNA were similar and the value calculated using the formula was 0.50 (i.e., within the range defined as no imbalance; > 0.3 < 0.7), is shown in Figure 2B.

LOH/NO LOH status was defined by the presence of at least two informative SNPs per chromosome arm with concordant results, one of which was located in a centromeric region and the other at a telomeric locus. Overall, we found that the average number of informative SNPs per sample was about six for both chromosome regions. As detailed in Table 1, more than two informative SNPs were available in all cases, with the exception of cases n. 29 and 36, showing only two informative SNPs on 19q chromosome arm. As reported in Supplementary Table 1, heterozygous/informative SNPs were evenly distributed along the chromosome arms for each sample, enabling discrimination between whole or partial chromosome arm deletions. We did not identify any partial deletions of 1p and/or 19q in the analyzed population, although the MS test has the potential to detect such changes.

**Table 1:** Molecular results obtained by MS assay for 1p/19q LOH, and IDH and TERT mutations

SAMPLE	HYSTOLOGICAL TYPE	WHO GRADE	LOH		IDH mutations	TERT mutations
			1p	19q		
1	ODG	II	LOH (3 SNPs)	LOH (6 SNPs)	*IDH1* R132H*	*TERT* G228A*
2	Anaplastic ODG	III	LOH (7 SNPs)	LOH (8 SNPs)	*IDH1* R132H*	*TERT* G228A*
3	ODG	II	LOH (10 SNPs)	LOH (5 SNPs)	*IDH1* R132H*	*TERT* G228A
4	Anaplastic ODG	III	LOH (6 SNPs)	LOH (7 SNPs)	*IDH1* R132H*	*TERT* G228A*
5	ODG	II	LOH (8 SNPs)	LOH (3 SNPs)	*IDH1* R132C*	*TERT* G228A*
6	ODG	II	LOH (6 SNPs)	LOH (4 SNPs)	*IDH1* R132H*	*TERT* G228A*
7	ODG	II	LOH (6 SNPs)	LOH (6 SNPs)	*IDH1* R132H*	*TERT* G228A
8	Anaplastic ODG	III	LOH (7 SNPs)	LOH (7 SNPs)	*IDH1* R132H*	*TERT* G250A
9	ODG	III	LOH (3 SNPs)	LOH (6 SNPs)	*IDH2* R172W*	*TERT* G228A
10	ODG	II	LOH (5 SNPs)	LOH (6 SNPs)	*IDH1* R132H*	*TERT* G228A
11	ODG	II	LOH (5 SNPs)	LOH (4 SNPs)	*IDH1* R132H*	*TERT* G250A
12	Anaplastic ODG	III	LOH (9 SNPs)	LOH (9 SNPs)	*IDH2* R172K*	*TERT* G228A
13	ODG	II	LOH (9 SNPs)	LOH (6 SNPs)	*IDH1* R132H	*TERT* G228A
14	ODG	II	LOH (7 SNPs)	LOH (7 SNPs)	*IDH1* R132H*	*TERT* G228A*
15	ODG	II	LOH (8 SNPs)	LOH (4 SNPs)	*IDH1* R132H	*TERT* G228A
16	ODG	II	LOH (9 SNPs)	LOH (7 SNPs)	*IDH1* R132H*	*TERT* G250A
17	Anaplastic AC	III	NO LOH (9 SNPs)	NO LOH (10 SNPs)	WT*	WT
18	AC	II	NO LOH (7 SNPs)	NO LOH (5 SNPs)	*IDH1* R132C	WT
19	Anaplastic AC	III	NO LOH (5 SNPs)	NO LOH (6 SNPs)	*IDH1* R132H*	WT
20	AC	II	NO LOH (8 SNPs)	NO LOH (5 SNPs)	*IDH1* R132H*	WT
21	AC	II	NO LOH (13 SNPs)	NO LOH (8 SNPs)	*IDH1* R132H*	WT
22	AC	II	NO LOH (6 SNPs)	NO LOH (3 SNPs)	*IDH1* R132H*	WT*
23	Anaplastic AC	III	NO LOH (6 SNPs)	NO LOH (3 SNPs)	*IDH1* R132H*	WT
24	AC	III	NO LOH (8 SNPs)	NO LOH (5 SNPs)	*IDH1* R132H*	WT
25	GBM	IV	NO LOH (6 SNPs)	NO LOH (4 SNPs)	WT*	*TERT* G250A
26	GBM	IV	NO LOH (7 SNPs)	NO LOH (6 SNPs)	WT*	*TERT* G228A
27	GBM	IV	NO LOH (8 SNPs)	NO LOH (8 SNPs)	WT*	*TERT* G228A
28	GBM	IV	NO LOH (5 SNPs)	NO LOH (7 SNPs)	WT*	*TERT* G250A
29	GBM	IV	NO LOH (5 SNPs)	NO LOH (2 SNPs)	WT*	*TERT* G228A
30	GBM	IV	NO LOH (9 SNPs)	NO LOH (7 SNPs)	WT*	*TERT* G228A
31	GBM	IV	NO LOH (4 SNPs)	NO LOH (5 SNPs)	WT*	*TERT* G228A*
32	GBM	IV	NO LOH (5 SNPs)	NO LOH (6 SNPs)	*IDH1* R132H*	WT
33	GBM	IV	NO LOH (8 SNPs)	NO LOH (4 SNPs)	WT*	*TERT* G228A
34	GBM	IV	NO LOH (8 SNPs)	NO LOH (5 SNPs)	WT*	*TERT* G250A
35	GBM	IV	NO LOH (8 SNPs)	NO LOH (6 SNPs)	WT*	WT*
36	GBM	IV	NO LOH (9 SNPs)	NO LOH (2 SNPs)	WT*	*TERT* G228A*
37	GBM	IV	NO LOH (6 SNPs)	NO LOH (6 SNPs)	WT*	*TERT* G228A*
38	GBM	IV	NO LOH (7 SNPs)	NO LOH (8 SNPs)	WT*	*TERT* G250A
39	GBM	IV	NO LOH (9 SNPs)	NO LOH (7 SNPs)	WT*	*TERT* G228A*
40	GBM	IV	NO LOH (7 SNPs)	NO LOH (4 SNPs)	WT*	*TERT* G250A*
41	GBM	IV	NO LOH (6 SNPs)	NO LOH (6 SNPs)	WT*	*TERT* G228A*
42	GBM	IV	NO LOH (5 SNPs)	NO LOH (5 SNPs)	WT*	*TERT* G228A
43	GBM	IV	NO LOH (3 SNPs)	NO LOH (7 SNPs)	WT*	*TERT* G228A*
44	GBM	IV	NO LOH (4 SNPs)	NO LOH (10 SNPs)	WT	*TERT* G228A
45	GBM	IV	NO LOH (7 SNPs)	NO LOH (6 SNPs)	WT*	WT
46	GBM	IV	NO LOH (4 SNPs)	NO LOH (3 SNPs)	WT*	WT*
47	GBM	IV	NO LOH (6 SNPs)	NO LOH (8 SNPs)	WT*	*TERT* G228A*
48	GBM	IV	NO LOH (6 SNPs)	NO LOH (4 SNPs)	WT*	*TERT* G228A*
49	GBM	IV	NO LOH (7 SNPs)	NO LOH (5 SNPs)	WT*	*TERT* G228A*
50	GBM	IV	NO LOH (5 SNPs)	NO LOH (3 SNPs)	WT*	*TERT* G228A

ODG, oligodendroglioma; AC, astrocytoma; GBM, glioblastoma.

The number of informative SNPs for LOH analyses is shown in parentheses for each sample.

*confirmed by Sanger sequencing.

**Figure 3:**
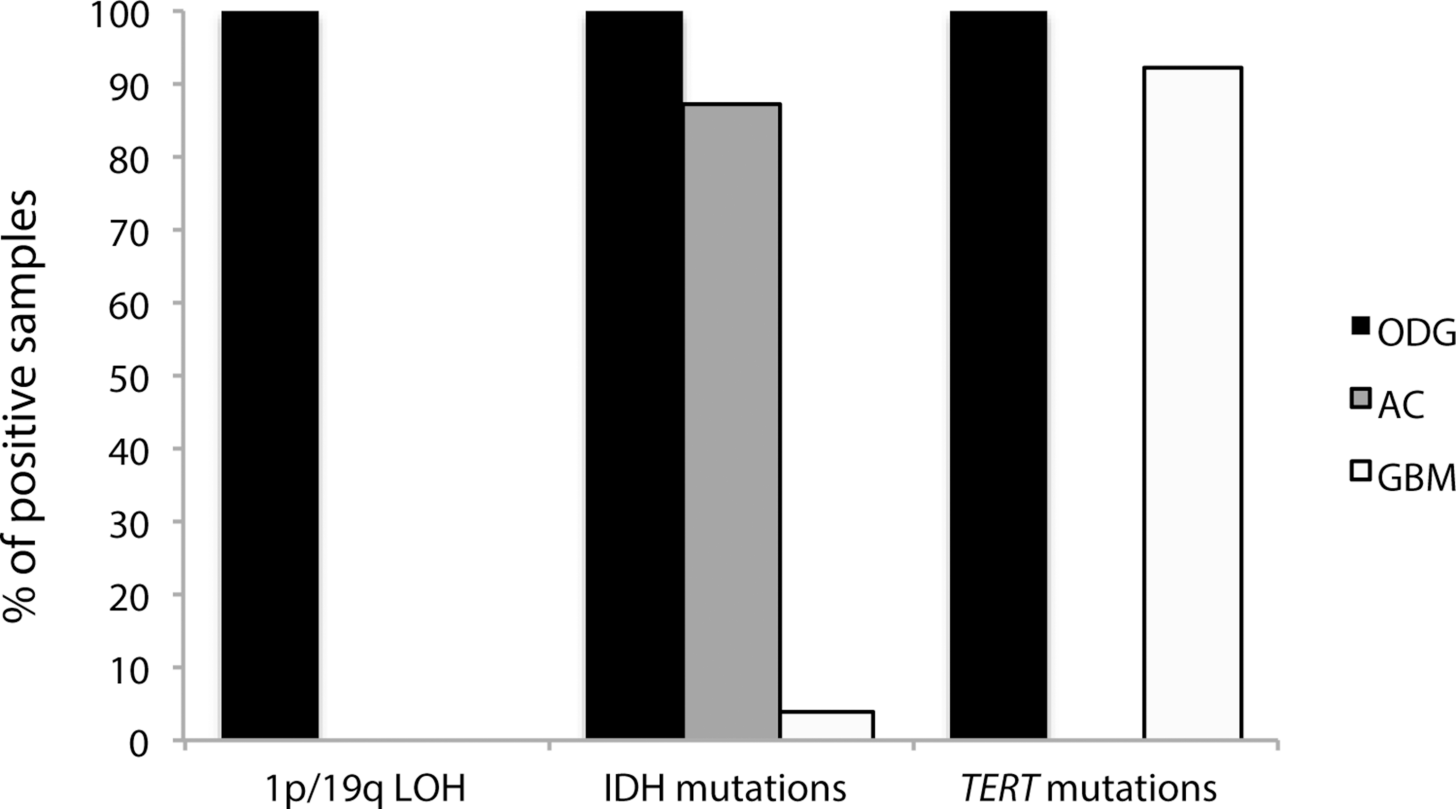
Distribution of the molecular markers analyzed in the three histological categories (ODG, AC and GBM) Anaplastic ODGs and anaplastic ACs were included in the ODG and AC classes, respectively.

Applying the diagnostic flowchart proposed in Figure 1, the molecular characterization by MS supported the histological diagnosis. Indeed, 1p/19q LOH is a typical feature of ODG and anaplastic ODG and, in our population, all 16 ODG samples (Table 1, cases n. 1–16) resulted positive for 1p/19q codeletion by MS. Conversely, all samples from tumors with other histotypes (AC and GBM) did not exhibit 1p/19q LOH or partial deletions of these chromosome arms (Table 1 and Figure 3).

**Figure 4:**
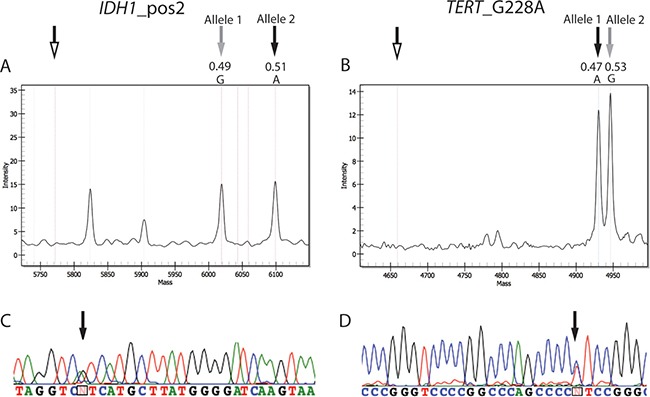
Spectra and electropheromgrams representative of IDH and TERT mutations (**A**) Mutation in position 2 (c.395G>A) of codon 132 of *IDH1* in case 4. (**B**) Mutation in position G228 (c.-124C>T) of the *TERT* promoter in case 37. Unfilled arrows, position of extension primers; gray arrows, wildtype alleles (Allele 1 for *IDH1* c.395 and Allele 2 for *TERT* c.-124); black arrows, mutant alleles. The allele frequencies of the wildtype and mutant alleles are indicated. Other peaks present in the spectra of *IDH1*_pos2 represent the allele signals of another SNP of the MS test. (**C** and **D)** display the results obtained by sequencing of the *IDH1* in case 4 and *TERT* in case 37, respectively. Black arrows correspond to the position of the mutated allele in the sequence.

As recommended by the 2016 CNS WHO [6], we also analyzed samples for mutations at codons 132 and 172 of *IDH1* and *IDH2*, respectively. In the MS panel, 24 out of 50 tumors contained one of these mutations. The most frequent mutation, found in 20 tumors (13/16 ODGs, 6/8 ACs, 1/26 GBMs), was *IDH1* c.395G>A (IDH1 R132H), in accordance with published data [7]; one ODG and one AC carried *IDH1* c.394C>T (IDH1 R132C) mutations, one ODG *IDH2* c.514A>T (IDH2 R172W), and one ODG *IDH2* c.515G>A (IDH2 R172K) (Table 1). The frequencies of the wildtype and mutated alleles generated using MS were always close to 0.5, implying that 1) the mutations were present in almost all analyzed cells; 2) the tumor cell content was close to 100%; and 3) the tumors were heterozygous for the mutations, in agreement with published data [5]. In particular, the average frequency of the *IDH1* allele encoding the R132H mutation was 0.52% ± 3% (data not shown). Figure 4A shows a representative spectrum from a mutated sample.

Finally, mutations in *TERT* promoter were found in 38 out of 50 samples. Thirty samples (13/16 ODGs, 17/26 GBMs) carried the c.-124C>T (*TERT* G228A) mutation and eight (three ODGs and five GBMs) the c.-146C>T (*TERT* G250A) mutation (Table 1), supporting the predominance of the G228A mutation in glioma [15]. Similar to the results of IDH gene analysis, the mutated allele of *TERT* was also highly represented (mean frequency: 0.59 ± 0.15); however, the high values of the standard deviation found among samples did not allow a precise quantification of the mutated allele, probably due to bias in the *TERT* amplification reaction. This could be caused by the high enrichment for G/C nucleotides in this promoter region, that prevents an optimal DNA amplification, as already suggested by another report [29]. This issue was not observed with any of the other assays performed in this study. Figure 4B illustrates representative results from *TERT* mutated samples.

Given the histological diagnoses of the analyzed samples, the results of our molecular analysis are in line with published reports [6]. Indeed, all ODGs were positive for both IDH and *TERT* mutations, seven out of eight ACs were IDH-mutant (cases n.18–24), and no AC samples had mutations in *TERT*. Finally, the IDH1 R132H mutation was found in only one out of 26 GBM samples (case n 32); conversely, the *TERT* mutations, which are frequent in GBM, were highly represented in our cases (22 out of 26 GBMs) (Figure 3).

### MS test validation

To validate the results of the analysis by MS (Table 1), we compared them with those obtained using reference methods. To test 1p/19q LOH, all 50 tumors were investigated by STR genotyping. The results obtained by this approach are detailed in Supplementary Figure 1A and are completely consistent with those from MS assay. Ten cases were also analyzed by FISH (cases n. 1, 2, 4, 5 and 7 positive and n. 24, 25, 26, 28 and 30, negative for 1p/19q LOH by MS, respectively). The FISH results are reported in Supplementary Figure 1B and FISH images, representative of 1p/19q codeletion and non-codeletion status, are depicted in Supplementary Figure 1C. FISH analyses unequivocally confirmed the MS results. Nevertheless, commercial FISH probes, commonly used to analyze 1p/19q codeletion, label only the telomeric portion of the 1p and 19q; on the contrary, the MS assay allows a fine mapping of chromosome deletions because it exploits a SNPs panel entirely covering 1p and 19q.

To further verify the efficiency of the MS test, four samples (samples nr 9 and 12, positive for LOH, and nr 29 and 37, negative for LOH) were analyzed by array-CGH. The results were concordant with those of MS, confirming once more that the MS test correctly detects the codeletion of the whole chromosome 1p and 19q arms.

Finally, Sanger sequencing was exploited to verify the hotspot mutations in *IDH1*, *IDH2* and *TERT* identified by MS. The sequencing analysis confirmed the results obtained by MS assay in all the investigated cases (marked by an asterisk in Table 1). Figures 4C and 4D displays the electropherograms of the respective MS spectra of two tumors (cases n. 4 and 37) with IDH1 R132H (Figure 4A) and *TERT* G228A (Figure 4B).

## DISCUSSION

The revision of the WHO classification of brain tumors integrated molecular with histological parameters for their diagnosis and made the analysis of genetic markers mandatory. Using MS technology, we propose a novel, reliable, and cost-effective test that combines analyses of the diagnostic markers 1p/19q LOH and IDH mutations, required by the 2016 CNS WHO, together with the detection of *TERT* promoter mutations, relevant to patient prognosis.

The 1p/19q LOH assay described in this paper genotypes 27 biallelic SNPs, carefully selected based on their frequencies in the general population and uniformly located across the entire spans of the chromosome 1p and 19q arms. The distribution of these SNPs enables discrimination between whole and partial chromosome deletions, and allows phenotype/genotype correlations, as previously reported [11, 35]. This advantage is shared by other techniques, including SNP-array and array-CGH, previously used to study 1p/19q status in gliomas [36] and, though these methods do not require a control DNA sample from the patient (unlike our technique), they are far less reliable when applied to samples with low DNA content or quality, such as FFPE samples. In routine diagnosis, indeed, application of methods such as SNPs or CGH array is challenged by the available amount of DNA, as these techniques require large quantities of starting material. By contrast, MS can accurately function using a very small amount of DNA (approximately 5 ng). FISH is an additional technique commonly exploited to study chromosome deletions in tumor tissues, because it does not require normal counterpart and can be performed also when the amount of neoplastic cells is low. Despite these advantages, FISH probes usually target solely the telomeric region of both chromosome arms, making the distinction between whole and partial chromosome deletions tricky. Furthermore, FISH is both laborious and time-consuming and in some cases the results are not conclusive and need PCR-based approach to study the LOH in order to complete the analysis [37].

A limitation of the MS test is that it requires a normal control sample to be applied. As a normal tissue we propose the use of patient blood sample, which could be easily obtained during the surgery and can be stored until the beginning of the analysis. However, when the blood sample is not available, the normal counterpart could be dissected from FFPE sample. If blood sample or other normal counterparts are not available, FISH or array-CGH should be performed. Another possible limitation of the MS test compared to FISH or array-CGH is that, similar to STRs analysis and also to NGS approaches [28–30], it reveals allelic imbalances but cannot distinguish between chromosome deletions or amplifications. Although 1p and 19q duplications are also possible features of glioma, concurrent duplications of both chromosome regions are a really rare event, thus the presence of concurrent imbalances is almost certainly due to a codeletion of both chromosome arms. The 1p/19q codeletion is precisely detected by MS (as demonstrated by our results) and it is currently the only chromosome imbalance with diagnostic value in glioma. However, when copy number aberrations are found in only one of the two chromosome arms, further analyses should be performed, to clarify the MS results.

In addition, the possibility of simultaneously genotyping a number of genetic loci allows the combination of all the required diagnostic analyses in one experiment, making MS the best choice to facilitate rapid diagnosis, also with limiting biological starting material. Indeed, using the MS test it is possible to analyze IDH mutations and 1p/19q status simultaneously, allowing the concomitant evaluation of the molecular markers deemed mandatory by the 2016 CNS WHO classification system.

Considering that MS would be simple to implement for new hotspot mutations, we extended our MS assay to evaluate mutations in the *TERT* promoter, which are not yet required by WHO, but are characteristic of both ODGs and GBMs and have recently been determined to have specific prognostic value in the molecular classification of gliomas [8, 22, 26]; moreover, *TERT* is also a potential therapeutic target, as recently demonstrated by the results obtained by Imetelstat treatment on various type of hematological and solid tumors [18, 19, 38, 39].

We did not include *ATRX* and *TP53* evaluation in the MS assay, since there are not hotspot mutations at these genes, and thus traditional sequencing or immunohistochemical staining would be more appropriate approaches for evaluation of these genes. *MGMT* promoter methylation status was also not included because analysis of DNA methylation requires a different MS protocol that cannot be combined using the genotyping approach.

In conclusion, the test described here represents a potential compromise between high-throughput technologies and cost- and time-effectiveness. The assay allows the concomitant evaluation of key markers of glioma characterization, comprising 1p/19q LOH status, and IDH and *TERT* mutations, conceivably in 2 working days, with limited cost per sample (about 80 euro per sample). The versatility of the MS technique makes it implementable with other new diagnostic markers; indeed, the MS tests could be also extended with the detection of other hotspot mutations, such as BRAF V600E, typical of few types of glioma, as epitheliod glioblastoma, or H3 K27M for diffuse midline gliomas, characteristic of pediatric patients [6].

Taken together, this approach may represent a step towards rapid, reliable, and cost-effective glioma diagnosis. The advantages and disadvantages of MS and the comparison with other techniques used to detect LOH are also detailed in Supplementary Table 2. Moreover, in this study, we demonstrated the feasibility of accurate detection of LOH using MS. Since also other chromosome deletions could become relevant for glioma diagnosis, such as 10q loss in GBMs or 9p loss in ODGs [20, 29], and, furthermore, deletions are also an important marker of genomic instability in other cancer types [40–42], the results using the MS approach in this study provide proof of principle for its future clinical application.

## MATERIALS AND METHODS

### Population

Fifty patients diagnosed with diffuse glioma from December 2013 to November 2016 at the Fondazione IRCCS Ca’ Granda, Ospedale Maggiore Policlinico di Milano were enrolled in the study on the basis of the availability of tumor and peripheral blood specimens. The study was approved by the Institutional Ethics Committee (Fondazione IRCCS Ca’ Granda, Ospedale Maggiore Policlinico no. 526/2015).

Twenty-four patients had LGGs (diffuse glioma, WHO grade II–III), including 12 ODGs, four anaplastic ODGs, five ACs, and three anaplastic ACs; the other 26 samples were high grade gliomas (HGGs; diffuse glioma, WHO grade IV), termed glioblastomas (GBMs) (Table 1). The median age at surgery was 53 years (range, 21 to 81 years); twenty-two patients were females and 28 males; forty-six patients underwent partial or total resection, while four patients underwent biopsy, due to the tumor location. This study focused on the development of a useful and updated molecular test for glioma diagnosis. Clinical aspects, such as overall survival and therapy response, were not considered.

### Biological specimens

Tumor DNA was extracted from FFPE sections using a Biostic FFPE tissue DNA isolation kit (MO BIO Laboratories, Carlsbad, CA, USA), following the manufacturer's instructions. FFPE tissue samples had previously been stained using hematoxylin and eosin and analyzed independently by two pathologists. The tumor cell content in all samples was at least 70%.

DNA from PBLs was isolated using the QiAMP DNA Mini Kit (Qiagen, Hilden, Germany), according to the manufacturer's protocol and used to test for the 1p/19q codeletion.

### MassARRAY procedures

Chromosome 1p/19q codeletion, and IDH and *TERT* mutations, were screened using a MassARRAY iPLEX platform (Agena Bioscience, San Diego, CA, USA), based on MALDI-TOF mass spectrometry. The results obtained using the MS assay were then validated by comparison with previous results obtained by STR genotyping or array-CGH.

### 1p/19q codeletion

**Figure 5:**
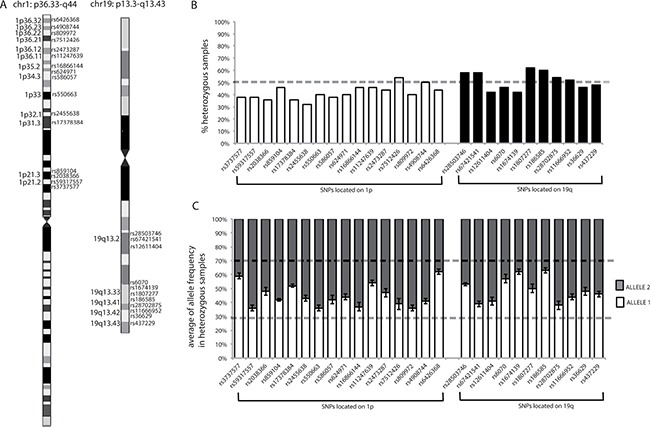
Chromosome location of SNPs, percentages of heterozygosity for each SNP, and mean allele frequencies for each SNP in heterozygous samples (**A**) SNP chromosome locations. Chromosome images originate from the UCSC database. The chromosome bands in which SNPs are located are indicated. (**B**) Graphical representation of the percentages of heterozygous samples for each SNP, calculated from the results obtained from the analysis of blood DNA samples from our population. Dotted line indicates 50%. (**C**) Graphical representation of mean allele frequencies for each SNP obtained from all heterozygous PBL samples in our population. The error bars indicate the standard deviation from the mean allele frequencies for each SNP. Dotted lines show the range of frequencies in which a sample was considered heterozygous (between 30% and 70%).

To evaluate chromosome 1p/19q codeletion in the tumor samples, LOH was tested for by analysis of a panel of 16 selected SNPs on 1p and 11 SNPs on 19q, both spanning the chromosome arms from their centromeric to their telomeric regions (Figure 5A and Supplementary Table 3). The homogeneous distribution of the SNPs along the two chromosome arms also provided the ability to discriminate between whole and partial chromosome arm imbalances. LOH assessment was performed by considering informative those SNPs heterozygous in PBL DNA, and comparing their genotypes in PBL DNA with those in tumor DNA.

Using data from the Ensembl database (http://www.ensembl.org/index.html), biallelic SNPs, with minor allele frequencies (MAFs) close to 0.5, were selected (Supplementary Table 3) since, in an ideal population, in accordance with Hardy-Weinberg Equilibrium, these SNPs would have a frequency of heterozygosity close to 0.5. Therefore, based on MAF values, the probability of identifying an individual homozygous for all of the SNPs was verified as being very low (< 1:20,000 for 1p and < 1:1,700 for 19q), thus ensuring that the MS assay had high sensitivity for detection of LOH.

Furthermore, the percentages of samples in our population heterozygous for each SNP were calculated, and all were confirmed to be around 50% (range, 32–62%) (Figure 5B), reflecting the assumption inferred from the MAFs in the general population.

To establish when a SNP could be considered heterozygous based on the MS results, the raw allele frequencies determined using the MassARRAY Typer software were exploited, which represented the percentage of each allele detected by the instrument for each specific SNP in the analyzed sample. Where Allele 1 had the lower molecular weight (appearing on the left of the spectrum) and Allele 2 the higher molecular weight (appearing on the right of the spectrum), allele frequencies ranged from 0 to 1, where 0 indicates the absence, and 1 the presence, of the observed allele only (i.e., homozygosity or hemizygosity). For example, if in a given sample the frequency of Allele 1 of SNP “x” is 0.54, the frequency of Allele 2 of this SNP will be 0.46; hence the sum of the allele frequencies is always 1. A SNP was verified as being considered heterozygous when the observed allele frequencies were between 0.3 and 0.7, as reported by the MassARRAY^®^ Typer 3.4 Software [43]. Furthermore, for each heterozygous SNP the variability of allele frequencies was investigated by evaluating the results generated using heterozygous PBL samples. As depicted in Figure 5C, the allele frequencies of each SNP generated by the instrument were stable among the normal control heterozygous samples, with standard deviations lower than 10%, confirming the reliability of the MS assay (for an example of allele frequency see Figure 2 or Figure 4, the intensity of the signal of each allele is reported on the y axis; from the intensity of each signal the MS software derives the respective allele frequency).

The following equation was used to quantitatively define the LOH status:

N2N1N2N1+T2T1

Where N_1_ and N_2_ are the frequencies of Allele 1 and Allele 2 found in PBL DNA and T_1_ and T_2_ are those of the corresponding alleles in tumor DNA. LOH was defined as detected when the value obtained using this formula was < 0.3 or > 0.7.

### IDH and *TERT* mutations

The analysis of codons 132 of *IDH1* (c.394C and c.395G; NP_001269316) and 172 of *IDH2* (c.514A, c.515G, and c.516G; NP_002159), and of the c.-124C>T (G228A) and c.-146C>T (G250A) *TERT* promoter (NP_937983) mutations, was included in the MS multiplexed assay. Negative and positive controls were included in each analysis; PBL DNA was used as a negative control while DNA samples from mutated tumors (previously identified by Sanger sequencing) were used as positive controls.

### MassARRAY reactions

PCR and extension primers were designed using Assay Design Suite v2.0, (https://www.agenacx.com/Logon.aspx?ReturnUrl=%2fTools), and multiplexed to obtain three PCR mixes. As indicated by the guideline procedures, a specific oligo tag sequence (5¢-ACGTTGGATG-) was added to the 5¢ end of each PCR primer to optimize the PCR reaction. Primers sequences are reported in Supplementary Table 3. Briefly, PCR amplification was conducted in 5 μl reactions containing 5–30 ng of DNA (depending on DNA availability), 100 nM PCR primers, 100 nM dNTP mix, PCR buffer, 25 mM MgCl_2_, and 5 U Taq DNA polymerase (Agena Bioscience, San Diego, CA, USA). The mixture was incubated as follows: 95°C for 2 min, 45 cycles of 95°C for 30 sec, 56°C for 30 sec, and 72°C for 60 sec, with a final extension step at 72°C for 5 min. Remaining unincorporated dNTPs were dephosphorylated and inactivated by treatment with 1.7 U of shrimp alkaline phosphatase at 37°C for 40 min and then 85°C for 5 min. Finally, the single base extension (SBE) reaction mix, including iPLEX Buffer Plus, iPLEX Termination Mix, Extension Primers mix, and iPLEX enzyme (Agena Bioscience, San Diego, CA, USA), was added to the PCR amplification products. The SBE reaction was carried out under the following conditions: 94°C for 30 sec; 40 cycles at 94°C for 5 sec [52°C for 5 sec and 80°C for 5 sec (repeated five times per cycle)]; and a final extension step at 72°C for 3 min. Samples were spotted on a SpectroCHIP (Agena Bioscience, San Diego, CA, USA), and finally analyzed by mass spectrometry. The spectral profiles generated by MALDI-TOF mass spectrometry were analyzed using Typer v.4.0 software (Agena Bioscience, San Diego, CA, USA).

### Evaluation of 1p/19q LOH by STR analysis

PCRs were performed using 100 ng of DNA from both tumor and PBL samples, and the products were analyzed by capillary gel electrophoresis using Gene Mapper software on an ABI 3130XL system (Applied Biosystems, Foster City, CA, USA). Six (D1S1592, D1S548, D1S2694, D1S2666, D1S1612, and D1S468) and three (D19S412, D19S596, and D19S206) STRs were used to investigate the presence of deletions on chromosomes 1p and 19q, respectively. The genomic locations of the STRs and the primer sequences used to amplify them are provided in Supplementary Table 4. LOH was assessed according to the peak-height ratio, as previously described [44]. In brief, the peak height derived from each allele amplified from both tumor and corresponding normal DNA was compared. The formula (T1/T2)/(N1/N2) was applied, where T1 and T2 are the peak heights of the alleles detected in tumor DNA, and N1 and N2 are the peak heights produced from PBL DNA. LOH was considered present when the result of the calculation was < 0.50. For values > 1.00, the ratio was converted to 1/[(T1/T2)/(N1/N2)] and, again, LOH was considered present if the resulting value was < 0.50.

### FISH analysis

FISH was performed in ten cases on 4-μm-thick sections of FFPE specimens, depending on the material availability. Five displayed 1p/19q LOH and five were without 1p/19q LOH, by MS and STR analyses. For each samples two slides were prepared, one for the detection of 1p deletion and the other one for 19q deletion. The sections were deparaffinized, treated with sodium thiocyanate and then digested with pepsin solution. Dual-color-probe hybridization was performed using ZytoLight SPEC 1p36/1q25 and 19q13/19p13 probes (ZytoVision, Bremerhaven, Germany). The spectrum-green-probes label the control regions 1q25 and 19p13 of each chromosome, while spectrum-red-probes mark the targets 1p36 and 19q13. Both probes for chromosomes 1 and 19 were denatured at 75°C for 10 minutes followed by an overnight hybridization at 37°C. Nuclei were counterstained with Leica mounting medium containing 4′,6-diamidino-2-phenylindole (DAPI) (Leica, Wetzlar, Germany) and examined under a Leica DM4000B Fluorescence microscope (Leica, Wetzlar, Germany) equipped with appropriate filters (DAPI, green and red). Signals of 100 non-overlapping nuclei with at least two control/green signals were enumerated for both chromosomes slides.

Interpretation of FISH images was performed accordingly to Ambros et al, 2001 [37]: normal pattern was defined by the presence of an equal number of control/green and target/red signals (i.e. control/target ratio: 2/2, 3/3, 4/4, etc), deletion pattern was characterized by the presence of at least two control/green signals but only one or zero target/red signals (i.e. control/target ratio: 2/1, 2/0, 3/1, etc); finally imbalance pattern was identified by the presence of more than 1 target/red signal (i.e. control/target ratio: 3/2, 4/2, 4/3, etc).

A sample was considered positive for 1p/19q codeletion when more than 50% of nuclei per chromosome arm displayed a typical deletion pattern [37, 45, 46].

### Array-CGH

In a subset of cases (two GBM and two ODGs), 1p/19q status was also confirmed by array-CGH. Array-CGH analysis was performed using 180 mer oligonucleotide probe technology (SurePrint G3 Human CGH 4 × 180K, Agilent Technologies, Santa Clara, CA, USA), according to the manufacturer's instructions. Raw data were generated using Agilent Feature Extraction and analyzed using Cytogenomics 3.0.4.1, with the ADM-2 algorithm (Agilent Technologies, Santa Clara, CA, USA). To improve the accuracy of the results, the Diploid Peak Centralization algorithm was also applied.

The aberration filter was set to detect a minimum of five consecutive probes/region, and the minimum absolute average log ratio (MAALR) was ± 0.25. A second analysis was run with a MAALR of ± 0.15 (again with a minimum number of five probes/region), to detect low level of mosaicism. Only copy number variants not already reported in the public database of genomic variants (http://projects.tcag.ca/variation/) were listed. Genomic coordinates are according to the build 37 assembly (March 2009) of the Human Genome Reference consortium (GRch37/hg19).

### IDH and TERT sequencing

The IDH and *TERT* hotspot mutations identified by MS were confirmed by sequencing in a subset of samples, based on DNA availability. The DNA regions spanning between the hotspot mutations in *IDH1*, *IDH2* and *TERT* genes were amplified using the primers reported in Supplementary Table 4. The PCR mixture of 20 μl volume contained 100 ng of DNA, 1X of PCR buffer, 1.5 mM MgCl_2_, 0.2 mM of each deoxyribonucleoside triphosphate, 0.5 μM of each primer and 0.2 units of AmpliTaq Gold DNA polymerase (Applied Biosystems, Foster City, CA, US). PCR program consisted of an initial denaturation performed at 95°C for 5 min, followed by 35 cycles of denaturation at 95°C for 20 sec, annealing for 20 sec and extension at 72°C for 30 sec, and a final extension step at 72°C for 5 min. PCR products were purified using the MinElute PCR Purification Kit (Qiagen, Hilden, Germany) and sequenced using the automated sequencer ABIPRISM 3130XL Genetic Analyzer (Applied Biosystems, Foster City, CA, US).

## SUPPLEMENTARY MATERIALS FIGURES AND TABLES







## Abbreviations

Array-CGH: Array comparative genomic hybridization
AC: Astrocytoma
CNS: Central nervous system
FFPE: Fixed-formalin paraffin-embedded
FISH: Fluorescence Is Situ Hybridization
GBM: Glioblastoma
IDH: *IDH1* and *IDH2* genes
LOH: Loss of heterozygosity
MAF: Minor allele frequency
MALDI-TOF: Matrix-assisted laser desorption ionization time-of-flight
MS: MassARRAY
ODG: Oligodendroglioma
SNP: Single nucleotide polymorphism
STR: Short tandem repeat
WHO: World Health Organization
