# Supplementary material for: Mass spectrometry-based assay for the molecular diagnosis of glioma: concomitant detection of chromosome 1p/19q codeletion, and IDH1, IDH2, and TERT mutation status
Source: Oncotarget. 2017 Jul 8;8(34):57134–48. doi: 10.18632/oncotarget.19103 (PMC5593631; doi:10.18632/oncotarget.19103)
Supplement: Supplementary file 1 [file oncotarget-08-57134-s001.pdf]

Mass spectrometry-based assay for the molecular diagnosis of glioma: concomitant detection of chromosome 1p/19q codeletion, and *IDH1*, *IDH2*, and *TERT* mutation status

SUPPLEMENTARY MATERIALS

A

1p STRs		SAMPLES WITH 1p/19q LOH															
		1	2	3	4	5	6	7	8	9	10	11	12	13	14	15	16
1p36.13	D1S1592		0,1	0,3	0,4		0	0,4	0,3		0,1			0,2	0,2	0,3	
1p36.23	D1S548			0		0,4		0,3		0	0	0,1	0	0	0,2		0
1p36.23	D1S2694		0						0	0							
1p36.23	D1S2666										0	0			0		0,2
1p36.23	D1S1612				0,3		0,2			0,1			0,2	0		0	0
1p36.32	D1S468	0			0,5	0,3							0	0,3	0,3		0
19q STRs																	
19q13.32	D19S412			0,4	0,2		0,1	0,2					0	0			
19q13.33	D19S596					0,1		0	0,3	0,1	0		0,1		0	0,4	0
19q13.41	D19S206	0	0	0	0,2	0	0,2	0,3	0	0	0,1	0	0				0,3

1p STRs		SAMPLES WITHOUT 1p/19q LOH																
		17	18	19	20	21	22	23	24	25	26	27	28	29	30	31	32	33
1p36.13	D1S1592	0,74	0,9	0,8	0,9			0,9	1	0,9	0,7			0,8	0,7		0,6	
1p36.23	D1S548			0,8						0,7		0,9	0,7		0,8		0,7	0,9
1p36.23	D1S2694		0,9				0,9	1			0,7	0,9	0,8					
1p36.23	D1S2666		0,9			0,9						0,8	0,8					
1p36.23	D1S1612	0,7	0,9	0,8			1					0,8				0,9		0,9
1p36.32	D1S468					0,8	0,9		0,9			1		0,8		0,8		0,8
19q STRs																		
19q13.32	D19S412	0,72	1	0,6		1			0,8	0,6	0,6	0,6	0,9		1	0,8	0,6	0,8
19q13.33	D19S596	0,68	0,8	0,6	0,9	0,8				0,5	0,6		0,9	0,6	1			0,8
19q13.41	D19S206	0,8		0,6		0,8	0,9	0,9			0,5	0,7	0,8	0,7	0,6	0,7		0,9

1p STRs		SAMPLES WITHOUT 1p/19q LOH																
		34	35	36	37	38	39	40	41	42	43	44	45	46	47	48	49	50
1p36.13	D1S1592			0,9	0,9	0,8		0,9	0,8		0,9	0,9					1	
1p36.23	D1S548			0,8		0,8	0,9	0,9	0,8	0,7	0,8	0,7	0,6		0,7		0,8	0,9
1p36.23	D1S2694				0,9			0,7	0,6	0,7		0,7	0,7	0,9		0,9	0,7	0,9
1p36.23	D1S2666					0,8		0,8			0,7	0,7	0,5				0,9	
1p36.23	D1S1612	0,9	0,9												1			
1p36.32	D1S468		1				0,8							1	0,9	0,7		
19q STRs																		
19q13.32	D19S412			0,9	0,9			0,9		0,9	0,9	1	0,9		0,8	0,9		
19q13.33	D19S596	0,9	0,6	0,9	0,9	1	0,5	0,9	0,9			0,9		0,6		0,9	0,7	0,8
19q13.41	D19S206	0,9	0,8				0,9		0,6	0,8	0,7		0,8	0,7	0,9		0,8	0,7

B

Sample	Chromosome	number of nuclei: NORMAL PATTERN	IMBALANCE	DELETION	Result
1	1p	20	3	77	1p/19q codel
	19q	15	2	83	
2	1p	17	19	64	1p/19q codel
	19q	18	24	58	
4	1p	20	6	74	1p/19q codel
	19q	19	3	78	
5	1p	18	2	80	1p/19q codel
	19q	23	2	75	
7	1p	15	7	78	1p/19q codel
	19q	25	0	75	
24	1p	85	6	9	Non codel
	19q	91	4	5	
25	1p	97	1	2	Non codel
	19q	93	1	6	
26	1p	96	4	0	Non codel
	19q	99	1	0	
28	1p	72	19	9	Non codel
	19q	86	10	4	
30	1p	86	7	7	Non codel
	19q	93	2	5	

C

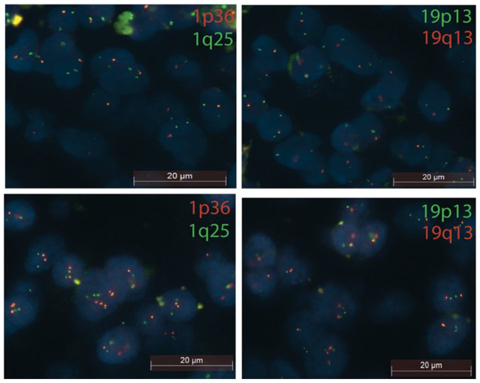

Supplementary Figure 1: The results obtained by STR genotyping of the fifty gliomas and by FISH on ten samples. (**A**) Detailed results of the heterozygous/informative STRs of each glioma sample included in the study. The first panel shows the 16 ODG cases positive for 1p/19q LOH by MS, while the second panel shows the remaining 34 cases without 1p/19q LOH. LOH was assessed applying the formula reported in Material and Methods and LOH was considered present when the result of the formula was < 0.50. (**B**) Summary of the FISH analysis on ten gliomas. The number of nuclei with each possible pattern is shown. In both chromosome 1 and 19, normal pattern is characterized by equal control/target signals, deletion pattern is present if the control signals are at least two and the target signal is 1 or 0, while imbalance pattern is a relative loss of target signals and is inconclusive to complete the analysis of 1p/19q codeletion. Only non-overlapping nuclei were considered and the cases were considered positive for 1p/19q codeletion if at least 50 of the 100 counted nuclei displayed a deletion pattern, that is the cut-off value suggested by the several authors [37, 45, 46]. Codel = codeletion. (**C**) Representative FISH images of case n. 4 with 1p/19q codeletion (top panels) and case n. 24, without 1p/19q codeletion (bottom panels). Nuclei were counterstained with DAPI, green spots mark the control signals for both chromosomes and red spots represent the target signals of both chromosomes. Sample 4 displays the typical deletion pattern (control/target signals = 2/1) at both 1p and 19q in the majority of nuclei, while sample 24 shows a normal pattern, characterized by equal control/target signals. Scale bars are reported.

Supplementary Table 1: Summary of informative SNPs in the 16 samples positive for 1p/19q LOH and the 34 samples negative for 1p/19q LOH. See Supplementary_Table_1

Supplementary Table 2: Comparison between MS and other techniques to reveal LOH

Technique	MS	STRs genotyping	FISH	array-CGH/ SNP-array	NGS
**LOH detection**	Yes	Yes	Yes	Yes	Yes
**Distinction between chromosome amplification and deletion**	No	No	Yes	Yes	Only with commercial kits or custom libraries, no one specific for glioma
**Point mutation detection**	Only hotspot mutations	No	No	No	Hotspot and novel mutations
**Multiplexing capability**	High	Low	High	No	High
**Time required**	2 days: -2 hrs for the bench work -30 min for the results analysis.	2 days: -1 hr for the bench work -30 min for the results analysis.	2 days: -4 hrs for the bench work -1 hr for the results analysis.	2 days: -1.5 days for the bench work -1 hr for the results analysis.	3 days: -1.5 days for the bench work -1 day for the results analysis (depending on the panel).
**-CE in vitro diagnostic commercial kit**	No	Yes	Yes	Yes	Genome-wide CNVs kits
**Custom assay**	Yes	Yes	Yes	No	Yes

FISH = Fluorescence In Situ Hybridization; CNVs = Copy Number Variations hr = hour; min = minute.

Supplementary Table 3: Primers and characteristics of genetic loci included in MS assay. See Supplementary_Table_3

Supplementary Table 4: DNA sequences and chromosome location of primers used for STRs, IDH and TERT mutations analysis

STR	LOCATION	REPEAT	SIZE	PRIMERS
D1S1592	1p36.13	CATT	236-244	FW: FAM-GGTGACAGGTATTGACTGCC<br>REV: TTGAGGGCAGAGATTGTCTC
D1S548	1p36.23	TATC	148-172	FW: HEX-GAACTCATTGGCAAAAGGAA<br>REV: GCCTCTTTGTTGCAGTGATT
D1S2694	1p36.23	CA	241-255	FW: HEX-AGGTCCAAGAAGCGGAG<br>REV: TCACGGGGTATTAAGTGG
D1S2666	1p36.23	CA	181-193	FW: FAM-AGCGAAACTCCATCTCAG<br>REV: TGCCTATGCTTTTGAGGT
D1S1612	1p36.23	TTCC	94-130	FW: HEX-TCCCATGCCAAAATTCTTAG<br>REV: GAAAGAAAGAGAAAGAAGGAAGG
D1S468	1p36.32	CA	173-191	FW: FAM-TTAACCGTTTTGGTCCTACC<br>REV: CTCTGACCAGCATTAAAGATTC
D19S412	19q13.32	CA	89-113	FW: FAM-GAATGAGACTCTGTCTCAAAACA<br>REV: ACCTCATGTGTCTCCTCCTT
D19S596	19q13.33	CA	213-221	FW: FAM-GAATCCGAGAGGTGGG<br>REV: GCCAGAGCCACTGTGT
D19S206	19q13.41	GT	103-146	FW: FAM-AGCCGAAGTCTTTTCACAAGAG<br>REV: TTCATCAAGTCTGTTCCAGCC

GENE	PrimerSequence (5′→3′)	PCR product lenght (bps)	Annealing Temperature (°C)
IDH1	FW: CGGTCTTCAGAGAAGCCATT<br>REV: CACATTATTGCCAACATGAC	129	57
IDH2	FW: AGCCCATCATCTGCAAAAAC<br>REV: CTAGGCGAGGAGCTCCAGT	150	57
TERT	FW: AGTGGATTCGCGGGCACAGA<br>REV: GCAGCGCTGCCTGAAACTCG	236	64

FW = Forward primer; REV = Reverse primer.
All the information reported are provided by Ensembl database (http://www.ensembl.org/index.html; accessed in December 2016).
